# Evaluation of Immunocompetent Urinary Tract Infected Balb/C Mouse Model For the Study of Antibiotic Resistance Development Using *Escherichia Coli* CFT073 Infection

**DOI:** 10.3390/antibiotics8040170

**Published:** 2019-09-28

**Authors:** Ashok Chockalingam, Sharron Stewart, Lin Xu, Adarsh Gandhi, Murali K. Matta, Vikram Patel, Leonard Sacks, Rodney Rouse

**Affiliations:** 1 Office of Translational Science, Office of Clinical Pharmacology, Division of Applied Regulatory Science U.S. Food and Drug Administration, Center for Drug Evaluation and Research, White Oak Federal Research Center, Silver Spring, MD 20993, USA; 2 Office of Medical Policy; U.S. Food and Drug Administration, Center for Drug Evaluation and Research, White Oak Federal Research Center, Silver Spring, MD 20993, USA

**Keywords:** CFT073 UPEC strain, Mouse UTI model, ampicillin, ciprofloxacin, fosfomycin, antibiotic resistance

## Abstract

Urinary tract infections (UTI) are common worldwide and are becoming increasingly difficult to treat because of the development of antibiotic resistance. Immunocompetent murine models of human UTI have been used to study pathogenesis and treatment but not for investigating resistance development after treatment with antibiotics. In this study, intravesical inoculation of uropathogenic *Escherichia coli* CFT073 in immunocompetent Balb/c mice was used as a model of human UTI. The value of the model in investigating antibiotic exposure on in vivo emergence of antibiotic resistance was examined. Experimentally infected mice were treated with 20 or 200 mg/kg ampicillin, 5 or 50 mg/kg ciprofloxacin, or 100 or 1000 mg/kg of fosfomycin. Ampicillin and ciprofloxacin were given twice daily at 8 h intervals, and fosfomycin was given once daily. Antibiotic treatment began 24 h after bacterial inoculation and ended after 72 h following the initial treatment. Although minimum inhibitory concentrations (MIC) for the experimental strain of *E. coli* were exceeded at peak concentrations in tissues and consistently in urine, low levels of bacteria persisted in tissues in all experiments. *E. coli* from bladder tissue, kidney, and urine grew on plates containing 1× MIC of antibiotic, but none grew at 3× MIC. This model is not suitable for studying emergent resistance but might serve to examine bacterial persistence.

## 1. Introduction

Urinary tract infections (UTIs) are one of the most common bacterial infections in humans and a major burden to healthcare systems. UTIs cause an estimated 11 million doctor visits in the US annually and over 150 million doctor visits globally, with an annual cost upward of 6 billion dollars (USD) [1]. Uropathogenic Escherichia coli (UPEC) cause up to 80% of UTIs and are believed to spread from the gastrointestinal tract to the bladder through contamination of the urogenital tract via the urethral opening. Other etiological agents of UTI include gram-negative (Klebsiella, Pseudomonas and Proteus) and gram-positive (Staphylococcus, enterococcus and group B Streptococcus) bacterial species [2].

The most commonly used antibiotics for the treatment of UTIs are ampicillin, ciprofloxacin, trimethoprim-sulfamethoxazole (TMP-SMX), fosfomycin, nitrofurantoin, cephalexin, and ceftriaxone. Although antibiotics have been used to treat UTIs for many years, the management of UTIs has become complicated by increasing rates of antimicrobial resistance. An in vitro study of antimicrobial resistance in urinary *E. coli* isolates among US outpatients from 2000 to 2010 showed greatest resistance increases for ciprofloxacin (3% to 17.1%), TMP-SMX (17.9% to 24.2%), and ampicillin (38.2% to 43.4%) [3]. Due to the increase in antibiotic resistance to other commonly prescribed antibiotics, in recent years, the older broad spectrum antibiotic, fosfomycin, has been increasingly prescribed for the treatment of multi-drug resistance (MDR) and non-MDR Enterobacteriaceae, including extended spectrum beta-lactamase (ESBL)- and carbapenemase- producing isolates [4]. However, increases in fosfomycin resistance have been reported in some countries [5,6]. Fortunately, the rapid in vitro emergence of resistance during fosfomycin monotherapy is rarely observed in vivo [7].

Increases in antimicrobial resistance in uropathogenic *E. coli* have led to the use of broad spectrum agents, combination antibiotic treatment, decreases in clinical cure rates, and higher risk of recurrence [8]. Measurements of Minimum Inhibitory Concentrations (MIC) along with genotypic testing have been used to investigate the development of resistance in the laboratory and clinical isolates following treatment with different antibiotics [3,9,10,11]. In real-world situations, antibiotic resistance in bacteria is rapidly acquired in the laboratory but appears more rarely observed in clinical isolates [12,13]. The susceptibility or resistance patterns in laboratory cultures might sometimes mislead or misrepresent clinical treatment outcomes [14,15,16,17]. Studies assessing mutant selection in a relevant immunocompetent physiological system might more accurately depict risk of as well as potential paths to in vivo resistance evolution.

Animal models, especially mouse models, are commonly utilized to assess the efficacy and pharmacokinetic/pharmacodynamic (PK/PD) relationships of antibiotics in urinary tract infections and to optimize the efficacy and minimize the cost and duration of clinical trials [18]. Limited studies have examined animal models as a primary approach for studying the development of antibiotic resistance [9,19,20,21]. To further examine the feasibility of an immunocompetent animal model as a model for investigating mechanisms of resistance development, the current study assessed the emergence of resistance in UPEC recovered from an immunocompetent Balb/c mouse UTI model experimentally infected with CFT073 UPEC and treated with ampicillin, ciprofloxacin, or fosfomycin.

## 2. Results

**Figure 1:**
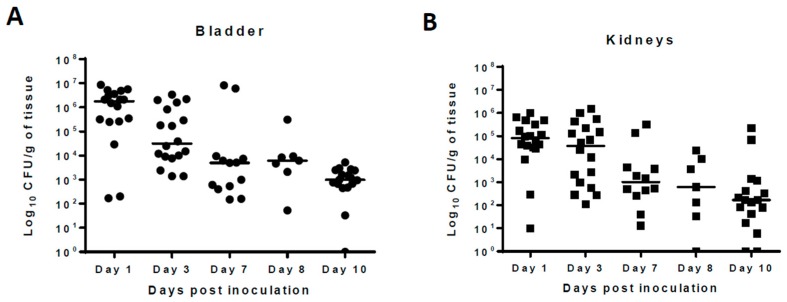

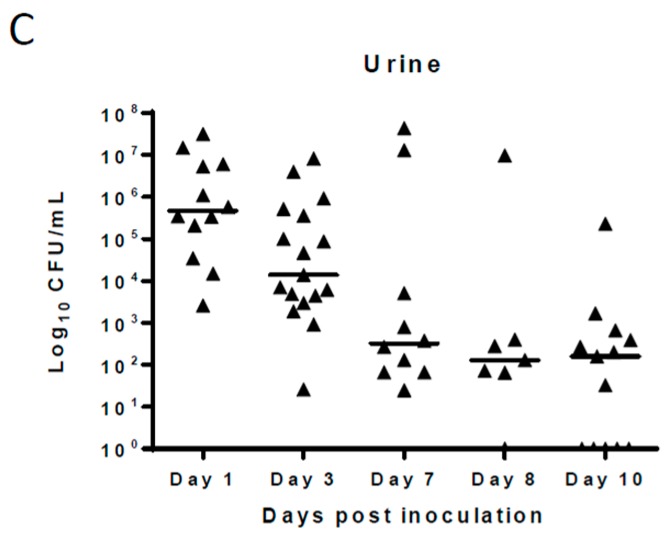
Natural history of *Escherichia coli* CFT073 strain urinary tract infections (UTI) in a Balb/c mouse strain. Mice were inoculated into urinary bladder with 10^8^ colony forming units (CFU) of *E. coli* by transurethral catheterization on day 0. Following infection, mice were sacrificed at day 1, 3, 7, 8, and 10 post-infection and the bacterial counts (number of CFU) from urinary bladder (per gm) (**A**), kidneys (per gm) (**B**), and urine (per mL) (**C**) are shown at different post-infection days. Value zero was plotted as the limit of detection. Solid horizontal line represents the median bacterial count for the groups. The data shown was pooled from three separate experiments done at different times; *n* = 18 for day 1 and 2, *n* = 13 for day 7, *n* = 7 for day 8, and *n* = 17 for day 10. For urine samples, the number of animals varies based on the presence of urine in the urinary bladder at the time of necropsy.

Pilot studies showed that following intravesical inoculation of Balb/c mice with 10^8^ cfu of UPEC, organisms persisted in the urinary bladder, kidneys, and urine samples 10 days after initial infection in the absence of antimicrobial treatment. The median bacterial counts at 24 h post-infection were 10^6^, 10^5^, and 10^5.5^ cfu/mL for urinary bladder, kidneys, and urine, respectively. The bacterial counts gradually declined over the course of infection and, at 10 days post-infection, stood at 10^3^ cfu/mL for urinary bladder and 10^2^ cfu/mL for kidney and urine samples. (Figure 1). No bacteria were recovered from the tissues or urine of any naïve mice included in the study. For this manuscript, all samples from which no bacteria were cultured were considered sterile.

**Figure 2:**
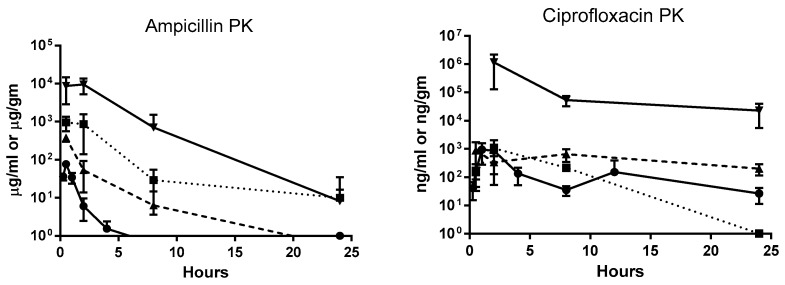

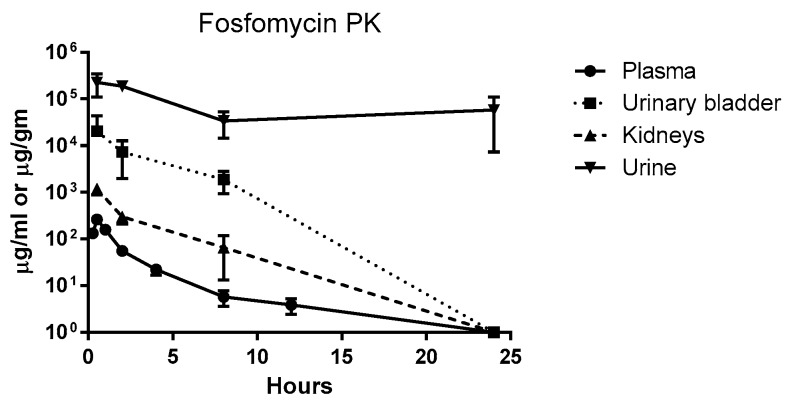
Single dose pharmacokinetics of ampicillin, ciprofloxacin, and fosfomycin antibiotics in female Balb/c mice. Groups of six mice were administered orally with 200 mg/kg of ampicillin, 50 mg/kg of ciprofloxacin and 500 mg/kg of fosfomycin. Plasma were sampled at 0, 15, and 30 min, 1, 2, 4, 8, 12, and 24 h time points. Urinary bladder, kidneys and urine were sampled at 0, 30 min, 2, 8, and 24 h time points during necropsy. Each symbol represents mean and standard deviation from six animals.

Single-dose pharmacokinetic (PK) studies were conducted for all three antibiotics (Figure 2). Ampicillin and fosfomycin concentrations peaked in plasma, urinary bladder, and kidneys 30 min after a single dose. Fosfomycin concentrations in urine also peaked at 30 min, but in ampicillin-treated mice, urine concentration did not reach a maximum until 2 h after dosing. Peak plasma ciprofloxacin concentrations were observed 1 h post administration. Urinary bladder and urine ciprofloxacin concentrations peaked 2 h after dosing, and in kidneys, maximum concentrations were reached 30 min post administration (Figure 2). In three-day dosing studies, all three antibiotics reached their highest plasma concentration 2 h post-administration in high dose–treated mice. Post-dosing, urinary bladder tissues showed highest antibiotic concentration at 2 h for ampicillin and fosfomycin and at 4 h for ciprofloxacin. Highest antibiotic concentration for kidney tissues was observed at 2 h for ampicillin and ciprofloxacin and at 4 h for fosfomycin. All three antibiotics were found in urine at high multiples of MIC across all time points. In bladder and kidney tissues, ampicillin and fosfomycin concentrations were several times higher than minimum inhibitory concentrations (1× MIC) 2 and 4 h after administration, respectively, whereas ciprofloxacin concentrations were above 1× MIC in these tissues throughout the study period (Table 1, Table 2, Table 3 and Table 4). The peak antibiotic concentrations in plasma, urinary bladder, kidneys and urine were different between single dose and multi-dose pharmacokinetic studies due to inclusion of earlier time points (0, 15, and 30 min, 1, 2, 4, 8, 12, and 24 h) in single-dose studies compared to multi-dose studies (24, 26, 28, 32, 48, and 72 h). Also fosfomycin dose concentrations were different for single-dose PK study (500 mg/kg) and high-dose studies (1000 mg/kg). As fosfomycin was cleared rapidly from urine as seen in single dose 500 mg/kg fosfomycin PK study, 1000 mg/kg was selected in the high dose experiment.

Mice treated with low dose ampicillin had consistently higher median bacterial counts in the urinary bladder, kidneys, and urine compared to high dose mice, although this difference was only significant at 48 h after treatment initiation in bladder tissue (Table 1). Median bacterial counts in high-dose groups were reduced 2 and 4 h post-treatment corresponding with times when the ampicillin concentrations in the urinary bladder and kidneys were higher than MIC of UPEC (Table 1). Most urinary bladder and kidney tissues in the high-dose ampicillin groups were sterile, while only urine samples after 72 h of treatment in the low-dose group were sterile (Table 1). No significant difference in the median UPEC counts were found in the urinary bladder, kidneys, and urine of mice treated and control groups after 72 h except in the kidney tissue of the low-dose group (Table 1).

There were no differences in median bacterial counts in urinary bladder, kidneys, and urine between mice treated with high- or low-dose ciprofloxacin except in the 48 h group. Urinary bladder tissue UPEC were eliminated completely from the urine after 48 h in mice treated with high-dose ciprofloxacin and after 72 h in most mice treated with low-dose ciprofloxacin (Table 2). The 72 h treatment and controls did not show any significant difference in the median UPEC counts in urinary bladder, kidneys, and urine either in the low- or high-dose ciprofloxacin experiments (Table 2).

For mice treated with fosfomycin for three days, significant differences in the median bacterial counts of the urinary bladder tissues were observed between high and low dose–treated groups. Although significant count differences were not seen in the kidney, the majority of the high-dose fosfomycin-treated mice had sterile kidneys, and none were sterile in the low-dose groups. In high-dose urine samples, UPEC were largely eliminated at all time points (sterile urine), while this was observed with less frequency in low-dose group urine (Table 3). As observed with ampicillin and ciprofloxacin studies, fosfomycin 72 h treatment and control groups did not show any significant difference in the median UPEC counts in the urinary bladder, kidneys, and urine (Table 3).

In the long term study, mice treated with fosfomycin showed persistence of UPEC in the bladder and kidneys even after three weeks of treatment. The median bacterial count in the urinary bladder and kidneys at the end of each of the three week treatments were similar, but the number of sterile mice increased in the second and third weeks whether in treated or control groups. Urine samples showed the presence of UPEC in the first week of fosfomycin treatment but bacteria in the urine were not detected after the second and third weeks of fosfomycin treatment. Control group urine samples showed the presence of UPEC after week one and in a few animals after week two, but UPEC were completely eliminated following the third week with no treatment (Table 4).

The resistant development of *E. coli* against antibiotics were screened on respective 1× MIC antibiotic media (Table 5). High and low dose ampicillin treatment yielded no 1× MIC UPEC colonies although one urine sample from a control group produced a single colony on 1× MIC media. A few UPEC colonies grew in 1× MIC (0.03 µg/mL) ciprofloxacin media from urinary bladder and kidney tissues in 24 and 72 h. low dose groups and from 28- and 32-h high dose groups. One urine sample in the 24 h high dose group grew UPEC at 1× MIC (64 µg/mL) fosfomycin media. Among all the samples screened for the long-term fosfomycin study, only one bladder sample at day 4 following 3 weeks of fosfomycin treatment grew a few UPEC colonies on 1× MIC (64 µg/mL) media (Table 5). The 1× MIC colonies did not show higher resistance on 3× MIC media. In this study, growth at 3× MIC was considered resistant. Thoughout the study no colonies were recovered that met this criterion.

**Figure 3:**
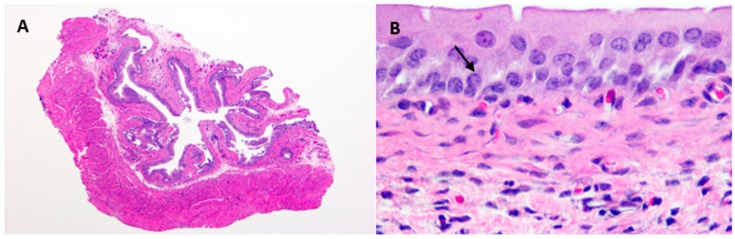

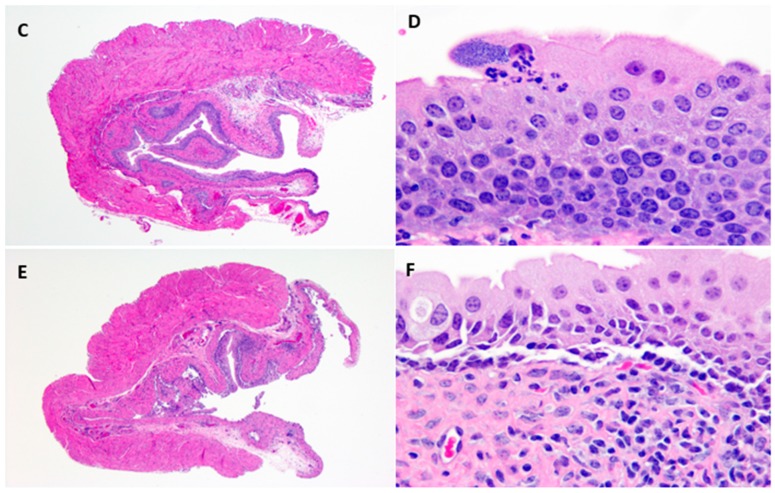
Representative photomicrographs of saline control (**A**,**B**), 1 DPI (**C**,**D**), and 10 DPI (**E**,**F**). Control urinary bladders exhibit a normal urothelium with minimal mucosal lymphocytes and edema. Intracellular bacterial communities (black arrow) admixed with neutrophilic infiltrates, marked urothelial hyperplasia, and mucosal edema with lymphocytic inflammation are observed at 1 DPI. At 10 DPI, urothelial hyperplasia is minimal and variable lymphohistiocytic mucosal infiltrates with rare neutrophils are still apparent. A, C, E @ 4× and B, D, F @ 60×. H&E stain. DPI = days post infection.

Hematoxylin and Eosin stained uninfected control urinary bladders exhibited a normal urothelium with minimal mucosal lymphocytes and edema (Figure 3A,B). *E. coli* –infected bladder tissue sections showed intracellular bacterial communities admixed with neutrophilic infiltrates, uroepithelial hyperplasia, and mucosal edema with lymphocytic inflammation as early as one-day post-infection (Figure 3C,D). However, by 10-days post-infection, with reduction in bacterial counts, pathological changes were minimal, with only slight urothelial hyperplasia and variable lymphohistiocytic mucosal infiltrates present (Figure 3E,F).

## 3. Discussion

A mouse model of human UTI was assessed for utility in understand the pharmacodynamics of antimicrobial therapy and the emergence of bacterial resistance. Rodents share much in common with humans, including immunological factors and anatomical features within the urinary tract. Murine models of human UTI have long been used to study UTI pathogenesis, assessment of treatment efficacy of antibiotics to a particular bacterial strain, or comparing the mutant and wild type bacterial strains for antibiotic efficacy [11,22,23,24]. Although the mouse model of UTI has been utilized to study the pathogenesis and treatment efficacy of antibiotics, none of the studies have explored the utility of this model for investigating resistance development in UTI infection after treatment with antibiotics. A few studies have examined the mouse model for emergent resistance in intestinal bacteria such as Klebsiella pneumoniae and Campylobacter jejuni in response to oral or subcutaneous exposure to fluoroquinolone or enrofloxacin. In the present study, the mouse model of UPEC CFT073 UTI was evaluated as a potential model for investigating the evolution of resistance development.

In the present study, ampicillin, ciprofloxacin, and fosfomycin treatment of UTI in mice reduced but did not eliminate tissue bacterial counts in any of the experiments regardless of dosing concentration or duration. Following a single dose, ampicillin and fosfomycin antibiotic concentrations in the urinary bladder and kidneys of treated mice exceeded the minimum inhibitory concentration during the first 4 h and 8 h post administration, respectively. By 24 h post treatment with ampicillin or fosfomycin, tissue antibiotic concentrations were below the MIC of UPEC. Ciprofloxacin concentrations exceeded the MIC of UPEC between ciprofloxacin dosing in urinary bladder, kidneys, and urine throughout the study. Although the antibiotic concentrations exceeded the MIC of UPEC during initial hours of ampicillin and fosfomycin administration and all hours of ciprofloxacin administration, UPEC were not eliminated completely but persisted at low levels in the urinary bladder and kidney tissues of infected mice. Histopathology of bladder tissues in our study further confirm the persistence of UPEC by forming intracellular bacterial communities (IBCs) inside the epithelial cells of urinary bladder (Figure 3D). Higher persistence in the bladder and the observation of intracellular bacteria suggest that UPEC may establish sequestered intracellular bacterial communities within the bladder epithelial cells where they are protected from host defenses and antibiotic exposure. These results are in agreement with an earlier study [25] that showed that a panel of 17 different antibiotics, representing seven distinct functional classes, did not completely eradicate UPEC UTI89 from the urinary bladder of infected mice and that only a few of the antibiotics (nitrofurantoin, ciprofloxacin, and sparfloxacin) were able to eliminate intracellular bacteria from the bladder epithelial cells in an in vitro cell culture assay. In another study, neither fosfomycin alone or in combination with cefoxitin eliminated bacteria completely from the kidneys of mice infected with CFT073-RR [11]. As noted in the above studies, UPEC were not completely eliminated from urinary bladder and kidneys even when the antibiotic pressure applied differed in antibiotic concentration and dosing duration. The present study identified no definitively resistant UPEC.

Treatment was more efficacious in urine where each of the these antibiotics is concentrated. Antibiotic concentrations in the urine of antibiotic-treated mice far exceeded the minimal inhibitory concentrations with all antibiotics at all time points. Urine samples were in most cases cleared of all bacteria, presumably due to much higher antibiotic concentrations in the urine than those achieved in bladder (1000× for ampicillin and fosfomycin) or kidney tissue (1000× for ampicillin, 25× for fosfomycin and 100× for ciprofloxacin). Other studies have shown the presence of higher antibiotic concentration in the mouse urine after administration of ampicillin, ciprofloxacin, and fosfomycin [26,27,28].

Antibiotic pressure on UPEC at very different concentrations and over different time periods did not reveal any emergent resistance or significant difference in bacterial persistence in urinary bladder and kidneys, which often did not differ much from persistence in control mice. Longer-term intermitant treatment of fosfomycin did not completely eliminate the bacteria from urinary bladder and kidneys of treated mice but also did not result in increased prevalence of antibiotic resistance. Investigation of resistance development in the laboratory or clinical isolates following treatment with different antibiotics have been examined by antimicrobial susceptibility testing and genotypic characterization [9,10,11]. Studies have shown that susceptibility or resistance patterns in laboratory cultures mislead or misrepresent clinical treatment outcomes [14,15,16,17]. The correlation between in vitro antimicrobial activity and the in vivo response to antibiotic treatment is affected by several factors including host defenses, the site and nature of infection, and the pharmacokinetics of antibiotics and its penetration into areas of infection. In vitro and immunocomprimised in vivo models interrogating antimicrobial resistance lack cellular factors and other antimicrobial small molecules found only in the immunocompetent in vivo environment that modulate the evolution of antibiotic resistance. A few studies have examined the mouse model for resistance development during or after treatment with antibiotics, but these studies were unable to identify resistant organisms to the drug used for treatment. Pultz et al. (2008) showed that treating mice subcutaneously with ciprofloxacin, levofloxacin, and moxifloxacin after orogastric administration of fluoroquinolone-resistant Klebsiella pneumoniae did not result in any emergence of fluoroquinolone-resistant gram-negative bacilli in the gut [20]. In another study, Inglis et al., (2018) used a mouse model to examine the impact of therapeutic administration of enrofloxacin on fluoroquinolone resistance development in *Campylobacter jejuni*. None of the *C. jejuni* isolates recovered from mice after subcutaneous or oral administration of enrofloxacin developed resistance to enrofloxacin and only modest shifts in the MIC of the isolates were observed [19]. Similarly the present study results indicate that this immunocompetent murine model of UTI is unsuitable to explore the emergence of antibiotic resistance following treatment but may be useful to investigate bacterial persistence.

In the present study, occasional growth was seen in a 1× MIC environment after intermittent or suboptimal duration of clinical or subclinical exposures to ampicillin, ciprofloxacin or fosfomycin. Due to the sporadic and infrequent nature of this growth and that growth was not duplicated at 3× MIC, these were not considered truly “resistant” and were not explored for genotypic changes in the bacteria. The inability to exacerbate emergent resistance suggests that resistance may be evolving similarly in this mouse model as it does clinically but that, as is the case clinically, this would be too infrequent and unpredictable to serve as a good model for studying emergent resistance. The protective effect of the immune competent urinary tract was demonstrated in the present study as the infected mice treated with excipients alone eliminated most of the UPEC at 72 h post-infection, similar to the antibiotic treated groups. This suggests that in most situations immune factors are responsible for resistance suppression in immune competent mice. The few colonies observed in the present study may have been mildly “resistant” due to genetic or physiological adaptations to antibiotic pressure in the micro-environment of urinary tract tissues or may not have been truly resistant if more rigorously tested. It is critical to note that non-resistant bacteria consistently persisted in urinary tract tissues, becoming a previously exposed reservoir of bacteria with a potential for emergent resistance.

## 4. Materials and Methods

### 4.1. Bacterial Strains, Media, and Chemicals

The clinical uropathogenic *Escherichia coli* (UPEC) isolate CFT073 purchased from ATCC (Manassas, VA, USA) was used for experimental infection in a UTI mouse model for antibiotic resistance development studies. Luria-Bertani (LB) broth and agar plates were used for the growth and selection of UPEC isolates. Ampicillin trihydrate (Sigma, St. Louis, MO, USA), Cipro 5% suspension (Bayer HealthCare Pharmaceuticals Inc., Wayne, NJ, USA), or Monurol (Fosfomycin tromethamine) (Forest Pharmaceuticals Inc., St. Louis, MO, USA) were used for the treatment of infected animals. Ampicillin was dissolved in 0.1 M HCl, ciprofloxacin (Microgranules: Hypromellose 3 cP, Magnesium stearate, Poly Acrylate Dispersion 30%, Polysorbate 20 and Povidone 25) was dissolved in cipro excipient (diluent: medium chain triglycerides, purified water, soy-lecithin, strawberry flavors 52312 and 54267, sucrose micronized), and Monurol was dissolved in water to create oral dosing solutions. Ampicillin sodium salt, ciprofloxacin HCl and phosphomycin disodium salt (Sigma, St. Louis, MO, USA) were purchased for creation of antibiotic containing LB agar plates. The laboratory specific MIC of ampicillin (4 μg/mL), ciprofloxacin (0.03 µg/mL), and fosfomycin (64 μg/mL) for the experimental strain were determined in-house by the in vitro broth dilution method following Clinical and Laboratory Standards Institute guidelines [10].

### 4.2. Animals

Female Balb/c mice 8–10 weeks old were purchased from Taconic Biosciences (Derwood, MD, USA) and were housed and maintained according to the Guide for the Care and Use of Laboratory Animals, 8th Edition, under an Institutional Animal Care and Use Committee approved protocol in the AAALAC accredited Animal Program of the White Oak Federal Research Center. Mice were provided with autoclaved feed (Prolab RM 3500, LabDiet, St. Louis, MO, USA) and were permitted food and water ad libitum. Animals were acclimated to the facility for six days before commencement of experiments. Individual mice were randomly assigned to treatment groups. At the initiation of each experiment, animals were socially housed according to facility standards in an animal cage with a wire mesh grid placed at the bottom, to avoid drug carry-over by coprophagic behavior.

### 4.3. Experimental Design

A pilot study was conducted to determine the UPEC persistence in the urinary bladder, kidneys and urine for 10 days post-infection (Days 1, 3, 7, 8 and 10) in a Balb/c mouse without any treatment. The single dose pharmacokinetics of ampicillin (200 mg/kg), ciprofloxacin (50 mg/kg) and fosfomycin (500 mg/kg) antibiotics in the mouse model were determined in pharmacokinetic studies (timepoints post-treatment—15 min., 30 min., 1 h, 2 h, 4 h, 8 h, 12 h, and 24 h). Based on these pilot studies, time points post-infection for UPEC estimation and dose and dosing interval for the antibiotics were selected for the main studies. High and low (10× lower than high dose) antibiotic doses were selected to study the resistance evolution under high and low antibiotic pressure environments. Antibiotic treatment duration was selected as a short-term treatment of three days for all three antibiotics (twice a day for ampicillin and ciprofloxacin; once a day for fosfomycin) and a long-term treatment of three weeks (short-term treatment protocol followed for three weeks) for fosfomycin only. The long-term fosfomycin treatment study was done to compare the long-term treatment pressure on the evolution of antibiotic resistance compared to short-term treatment pressure.

Based on these pilot and other prior studies, six mice per group were deemed minimally sufficient to demonstrate a difference in plasma concentrations between treatment groups. This difference was a factor in applying variable antibiotic pressure to the bacteria. Each experiment was limited to a total of 64 mice due to practical limitations to the number of mice that could be infected and the desired dosing time and sample collection schedules maintained. Mice were assigned to groups on the time point groups selected based on pharmacokinetics for each antibiotic experiment. This made the number of animals per group either six for an antibiotic requiring more time points or nine for an antibiotic with a longer half-life. Based on pharmacokinetics, the study included 2 and 4 h groups for all post ampicillin administration days and 2, 4, and 8 h groups on only the first day of post ciprofloxacin and Fosfomycin administration. To keep the animal numbers constant, the follow-on low dose antibiotic experiments included six animals per group.

For short-term high-dose studies (three days) with ampicillin, ciprofloxacin or fosfomycin, either six or nine female Balb/c mice were randomly assigned to the treatment groups. The low dose studies (3 days) included six mice to the treatment groups. Groups with 24, 48, and 72 h of antibiotic treatment were included in low and high dose treatment studies. As the focus of these studies was on resistance development of UPEC in vivo after antibiotic treatment, only one group of infected mice treated with excipient (solvent used for creating dosing solutions) was included as a control in each experiment. This control group was assessed 72 h after initial excipient exposure. Mice from antibiotic treatment and excipient control groups were given either antibiotics or excipient at the same dosing frequency for 1, 2, or 3 days beginning 24 h after infection. Groups of mice were sacrificed, and samples were collected either 24 h, 48 h, or 72 h after initiation of antibiotic treatment. High and low dosing concentration for experiments were ampicillin (200 and 20 mg/kg), ciprofloxacin (50 and 5 mg/kg), and fosfomycin (1000 and 100 mg/kg). A naïve (*n* = 4) group was included in each experiment to assess the sterility of the urinary tract tissues. To roughly compare multi-dose versus single dose pharmacokinetic parameters during high dose treatment experiments, groups were sampled at 2 h and 4 h following each day’s initial treatment with ampicillin and 2 h, 4 h, and 8 h following the second day’s initial treatment with ciprofloxacin and fosfomycin.

**Figure 4:**
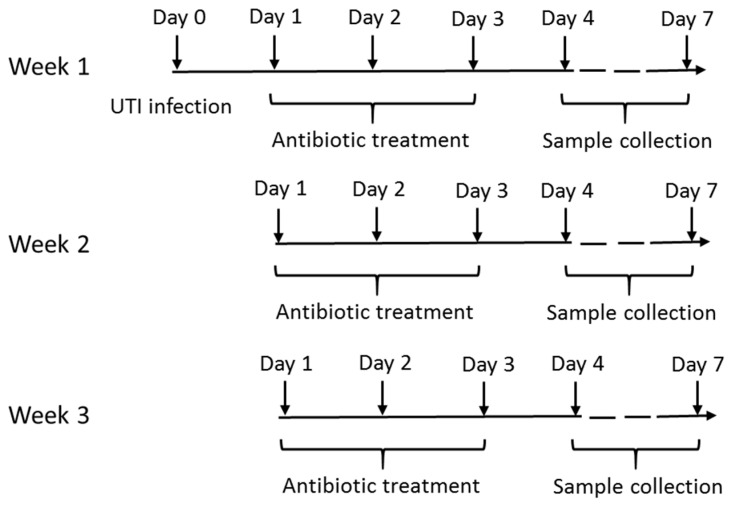
The long term study with repeated courses of fosfomycin (500 mg/kg) showing the infection, treatment and sample collection schedules.

A single long-term study looking at repeated courses of Fosfomycin was conducted. Twenty-four hours after experimental infection with UPEC, mice experienced the previously described three-day course of fosfomycin (500 mg/kg/day) or excipient that was repeated each week for three weeks. To avoid complete elimination of bacteria from the urinary tract and to provide longer antibiotic exposure for resistance development, a 500 mg/kg fosfomycin dose was selected for the long-term study. Urine and tissues from bladder and kidneys were sampled from groups 24 and 96 h after the last treatment each week. The infection, treatment, and sample collection schedules for the long-term study are shown below (Figure 4).

### 4.4. Murine Ascending Urinary Tract Infection

Eight- to 10-week-old immunocompetent female Balb/c mice were used to create a previously described [29] unobstructed ascending UTI mouse model with slight modification. The rat carotid artery polyurethane catheter (Cat. No. C19PU-RCA1301, Instechlabs, PA, USA) was used instead of PE10 catheter. The non-rounded end of the catheter was cut with sterile scissor to the length of a 30G ½ inch needle plus 1–2 mm. The catheter was inserted into the needle with the rounded end facing outside the needle for catheterization. The bacterial inoculum was prepared by overnight incubation at 37 °C in LB broth followed by re-inoculation in fresh LB broth. The bacterial cells were centrifuged at 5000 rpm for 10 min., and the pellets were dissolved in 10 mL of sterile phosphate buffered saline (PBS) and cell densities were adjusted to a final optical density OD_600_ of 1 to 1.1. This optical density corresponded to a cell density of between 8 × 10^8^ to 2 × 10^9^ colony forming units (CFU)/mL. Bacterial suspensions were aliquoted and maintained on ice until used. Urinary bacterial cystitis was induced by injecting 50 µL (approximately 4 × 10^7^ to 10^8^ CFU) of inoculum into the bladder by trans-urethral catheterization under isoflurane anesthesia. To confirm densities of viable *E. coli* cells, the inoculum was diluted 10-fold serially (10^−1^ to 10^−5^ dilutions), 100 μL of each dilution was spread in triplicate onto LB agar, incubated at 37 °C, and the number of *E. coli* colonies were counted after 16–18 h. of incubation.

### 4.5. Drug Administration

Antibiotics and excipients were administered via oral gavage 24 h after UTI infection using 18G stainless-steel gavage needles attached to 1 mL tuberculin syringes. Antibiotics were administered for three consecutive days in a total volume of 150 to 220 µL twice daily for ampicillin and ciprofloxacin and once daily for fosfomycin. Twice a day dosing was separated by an 8 h interval. Correspondingly the infected control mice were given 0.1 M HCl, cipro excipient or water in similar volumes as the ampicillin, ciprofloxacin, and fosfomycin treatment groups, respectively.

### 4.6. Sample Collection and Preparation

Mice were anesthetized by isoflurane general anesthesia, and a midline incision was made after spraying the skin with 70% ethanol. The urinary bladder was located and grasped by the neck with curved forceps. Urine samples were collected with a 27ga needle attached to a 1 mL syringe. Blood samples were collected by cardiac puncture and placed in 1 mL EDTA tubes. Death was assured by cervical dislocation followed by placement of aseptically collected urinary bladder and kidney samples in sample tubes containing 1.0 and 0.8 mL PBS, respectively. Urinary bladder and kidney samples were weighed upon collection, and all samples were kept on ice until processed. Blood samples were spun at 10,000 rpm for 15 min. at 4 °C, and resultant plasma samples were stored at–20 °C until analyzed for antibiotic concentration. The urinary bladder and kidney samples were homogenized with tungsten carbide beads in a Tissue Lyser apparatus (Qiagen, Carlsbad, CA, USA) and kept at 4 °C until estimation of susceptible and resistant UPEC and measurement of antibiotic concentration. The samples were first analyzed for the total and resistant UPEC population and then analyzed for the antibiotic concentration. If the samples were not analyzed for antibiotic concentration immediately after bacterial estimation, the samples were stored at −20 °C until analysis.

### 4.7. Estimation of Total and Resistant UPEC Population

The bacterial count in urinary bladder, kidney, and urine samples was quantified by spotting 50 µL of a series of 10-fold dilutions in LB broth or undiluted samples in triplicate on LB agar plates. The plates were incubated at 37 °C for 16–18 h, and the total UPEC population estimated and expressed as log_10_ CFU/mL. For preliminary assessment for antibiotic resistant UPEC, the samples were spotted onto LB agar plates containing 1× MIC of antibiotic incubated at 37 °C for 16–24 h. Samples with no growth were incubated for an additional 24 hr to check for slow growing resistant phenotypes. Colonies observed in the 1× MIC antibiotic plates were re-streaked on 1× MIC antibiotic plates to confirm the 1× MIC resistant phenotype and on 3× MIC plates to further evaluate resistance. In parallel, the 1× MIC plate was streaked with wildtype CFT073 *E. coli* to show no growth.

### 4.8. Estimation of Antibiotics in Urinary Tract Tissues, Urine, and Plasma

Urinary bladder, kidney tissues, and urine samples not used for estimating UPEC populations were stored either at 4 °C or −20 °C and used for measuring antibiotic concentrations. Blood samples collected in EDTA tubes were spun at 10,000 rpm for 15 min. at 4 °C, and the resulting plasma samples were stored at −20 °C until analyzed for antibiotic concentration. Ciprofloxacin was quantified in urine and urinary tissues by LC-MS/MS as previously described [30]. Fosfomycin and ampicillin quantifications were similarly done as previously reported [31,32], with slight modifications. For plasma and urinary tissues, samples were extracted with solvent in the ratio of 1:5 (matrix: precipitation solvent). For urine, samples were diluted 10× with water before extraction. All the other chromatographic and mass spectrometric conditions remained the same.

### 4.9. Histopathology

One half of bladder tissues from UTI infected and excipient control mice were collected for histopathological analysis. Gross observations were made, and subsequently tissues were fixed in 10% neutral buffered formalin for 48–72 h. Tissues were processed through graded alcohols, xylene and paraffin on an ASP300S tissue processor (Leica Biosystems, Buffalo Grove, IL, USA) and embedded into paraffin blocks. Then, 4 µm tissue sections were cut, mounted on glass slides, and stained with hematoxylin and eosin (H&E) (SelecTech; Leica Biosystems). The slides were examined by a veterinary pathologist.

### 4.10. Statistical Analysis

All data collected were in triplicates of each experiment, and the results are presented as median bacterial counts, range of bacterial counts, and number of sterile animals out of total number of animals. The statistical analysis between treatments as well as treatment and control groups were carried out using student’s t-test (two tailed) in GraphPad Prism 6 software. Statistically significant results were indicated as *p* < 0.05 and *p* < 0.01.

## 5. Conclusions

The present study assessed the feasibility of an immunocompetent Balb/c mouse model for investigating mechanisms of resistance development in UTI infection after treatment with antibiotics. After intermittent or suboptimal duration of clinical or subclinical exposures to ampicillin, ciprofloxacin, or fosfomycin, the study yielded sporadic and infrequent growth of *E. coli* in a 1× MIC environment that could not be duplicated at 3× MIC, which was considered the minimal threshold for resistance in this study. The immunocompetent murine model’s inability to demonstrate emergent resistance suggests that resistance may be evolving similarly in this mouse model as it does clinically, which would be too infrequent and unpredictable to serve as a good model for studying emergent resistance. However, this model could be used to study bacterial persistence in urinary tract tissues, which was observed consistently throughout the study with each of the antibiotics.

## Figures and Tables

**Table 1:** Ampicillin concentration and its effect on viable organisms in the urinary tract tissues (urinary bladder, kidneys, and urine) of mice infected with *E. coli* strain CFT073.

	Ampicillin Concentrations (µg/mL) and Bacterial Counts (cfu/gm or mL) ^@^									
Time (h)	Plasma	Bladder			Kidneys			Urine ^#^		
	Amp(µg/mL) High Dose	Amp (µg/mL) High Dose	Median Log_10_ cfu/gm		Amp (µg/mL) High Dose	Median Log_10_ cfu/gm		Amp (µg/mL) High Dose	Median Log_10_ cfu/mL	
			Range			Range			Range	
			(No. Sterile/Total)			(No. Sterile/Total)			(No. Sterile/Total)	
			High Dose	Low Dose		High Dose	Low Dose		High Dose	Low Dose
2	6.4 ± 3.8	217.4 ± 127.3	4.3	-	69 ± 46.7	3.6	-	2428 ± 928.1	5.2	-
			(2.8–5.2)			(2.8–4.6)			(2.3–5.6)	
			(0/6)			(0/6)			(0/5)	
4	1 ± 0.2	0.1 ± 0	4.6	-	14 ± 7.2	3.4	-	395 ± 172.5	5.1	-
			(2.9–5.2)			(3.0–4.1)			(4.9–5.9)	
			(0/6)			(0/6)			(0/5)	
24	0.3 ± 0.2	0.1 ± 0	3.8	5.5	0.1 ± 0	3.6	4.8	173.3 ± 145.6	4.3	4.7
			(3.3–4.2)	(3.4–5.9)		(1.6–4.4)	(4.4–5.9)		(2.3–4.8)	(3.0–6.2)
			(0/6)	(0/6)		(0/6)	(0/6)		(0/6)	(0/5)
26	24.5 ± 9.6	972.1 ± 818.5	3.2	-	203 ± 97.1	2.8	-	9044 ± 2933	2.2	-
			(2.4–3.7)			(1.3–3.7)			(1.3–2.5)	
			(0/6)			(0/6)			(2/4)	
28	8.3 ± 3.9	495 ± 409.7	2.9	-	73.9 ± 22.4	2.3	-	6356 ± 2425.4	3.0	-
			(1.4–3.0)			(1.4–3.2)			(2.3–4.1)	
			(1/6)			(0/6)			(0/3)	
48	0.1 ± 0.1	0.1 ± 0	2.8	4.3 *	0.1 ± 0	3.5	3.7	84.9 ± 33.7	2.0	4.1
			(2.3–3.3)	(3.6–4.7)		(2.8–4.0)	(2.0–4.8)		(-)	(3.0–4.3)
			(0/6)	(0/6)		(0/6)	(0/6)		(4/5)	(0/5)
50	31.8 ± 7.7	2180.1 ± 1977	2.3	-	298 ± 74.7	1.1	-	7010 ± 1885.7	2.5	-
			(2.2–2.6)			(0.8–1.4)			(2.0–2.7)	
			(1/6)			(1/6)			(4/6)	
52	18.6 ± 4.2	983.5 ± 356.3	2.3	-	203.9 ± 61.7	2.6	-	10338 ± 3974.2	2.3	-
			(0.8–3.4)			(1.7–2.8)			(2.3–2.3)	
			(0/6)			(4/6)			(2/5)	
72	0.2 ± 0.1	0.1 ± 0	2.1	3.6	0.1 ± 0	2.5	2.7	103 ± 55.1	2.2	2.3
			(0.8–3.4)	(2.1–3.8)		(1.1–3.6)	(1.9–4.0)		(2.0–2.3)	(2.0–3.3)
			(4/6)	(0/6)		(3/6)	(0/6)		(4/6)	(2/6)
Control	<LLOQ ^ϕ^	<LLOQ	2.6	4.1	<LLOQ	3.2	3.8 **	<LLOQ	2.7	3.7
			(1.4–3.5)	(4.0–4.2)		(2.1–5.2)	(2.6–5.7)		(2.6–2.8)	(2.6–5.0)
			(1/6)	(0/6)		(0/6)	(0/6)		(3/5)	(0/6)

^@^ Ampicillin MIC for CFT073 *E. coli* is 4 µg/mL. # For urine samples, the number of animals varies from 1–6 based on the presence of urine in the urinary bladder at the time of necropsy. ^ϕ^ LLOQ–Lower limit of quantification. * *p* < 0.05, bladder median UPEC cfu for 48 h high- and low-dose treatment groups; Student’s paired t-test. ** *p* < 0.01, kidneys median UPEC cfu for low-dose 72 h treatment and end of treatment control groups; Student’s paired t-test.

**Table 2:** Ciprofloxacin concentration and its effect on viable organisms in the urinary tract tissues (urinary bladder, kidneys, and urine) of mice infected with *E. coli* strain CFT073.

	Ciprofloxacin Concentration (µg/mL) and Bacterial Counts (cfu/gm or mL) ^@^									
Time (h)	Plasma	Bladder			Kidneys			Urine ^#^		
	Cipro (µg/mL) High Dose	Cipro (µg/mL) High Dose	Median Log_10_ cfu/gm		Cipro (µg/mL) High Dose	Median Log_10_ cfu/gm		Cipro (µg/mL) High Dose	Median Log_10_ cfu/mL	
			Range			Range			Range	
			(No. Sterile/Total)			(No. Sterile/Total)			(No. Sterile/Total)	
			High Dose	Low Dose		High Dose	Low Dose		High Dose	Low Dose
24	0.1 ± 0	223.7 ± 106.4	3.3	4.6	4.2 ± 3.7	1.9	3.4	198.5 ± 100.8	2.0	4.4
			(1.4–3.7)	(3.3–5.6)		(1.3–2.2)	(2.5–4.7)		(-)	(2–5.6)
			(0/8)	(0/6)		(3/8)	(0/6)		(4/5)	(0/6)
26	1.0 ± 0.2	705.7 ± 604.5	0.9	-	72.8 ± 66.5	2.1	-	965.4 ± 48.6	2.0	-
			(0.8–2.4)			(1.1–2.4)			(-)	
			(4/9)			(7/9)			(6/7)	
28	0.9 ± 0.1	796.1 ± 271.2	2.1	-	59.9 ± 85	2.0	-	NA	0	-
			(1.6–3.0)			(1.3–2.7)			(-)	
			(2/8)			(3/8)			(3/3)	
32	0.2 ± 0.1	495.7 ± 281.6	2.2	-	8.4 ± 5.9	2.4	-	451.4 ± 112	1.0	-
			(1.4–2.7)			(0.8–3.3)			(-)	
			(4/8)			(3/8)			(6/7)	
48	0.1 ± 0	173.9 ± 14.3	2.3	4.4 *	5.1 ± 3.7	2.9	2.3	378.9 ± 199.5	0	4.7
			(1.5–3.0)	(3.5–4.7)		(1.7–3.3)	(0.8–4.1)		(-)	(2.5–5.0)
			(0/8)	(0/6)		(3/8)	(0/6)		(6/6)	(1/5)
72	0.1 ± 0	160 ± 18.5	2.8	3.3	1.8 ± 0.6	2.1	2.3	237.3 ± 77.1	0	3.8
			(0.8–3.5)	(2.7–3.8)		(1.1–2.8)	(1.6–3.7)		(-)	(-)
			(0/9)	(0/6)		(3/9)	(0/6)		(7/7)	(5/6)
Control	<LLOQ ^ϕ^	<LLOQ	1.7	5.0	<LLOQ	4.3	2.1	<LLOQ	2.6	4.1
			(1.7–2.7)	(1.3–4.5)		(1.4–4.6)	(1.1–4.3)		(2.4–2.7)	(2.5–5.5)
			(0/3)	(0/6)		(1/3)	(0/6)		(0/2)	(1/6)

^@^ Ciprofloxacin MIC for CFT073 *E. coli* is 0.03 µg/mL. # For urine samples, the number of animals varies from 1–9 based on the presence of urine in the urinary bladder at the time of necropsy. ^ϕ^ LLOQ–Lower limit of quantification. * *p* < 0.05, bladder median UPEC cfu for 48 h high- and low-dose treatment groups; Student’s paired t-test.

**Table 3:** Fosfomycin concentration and its effect on viable organisms in the urinary tract tissues (urinary bladder, kidneys, and urine) of mice infected with *E. coli* strain CFT073.

	Fosfomycin Concentration (µg/mL) and Bacterial Counts (cfu/gm or mL) ^@^									
Time (h)	Plasma	Bladder			Kidneys			Urine^#^		
	Fosfo (µg/mL) High Dose	Fosfo (µg/mL) High Dose	Median Log_10_ cfu/gm		Fosfo (µg/mL) High Dose	Median Log_10_ cfu/gm		Fosfo (µg/mL) High Dose	Median Log_10_ cfu/mL	
			Range			Range			Range	
			(No. Sterile/Total)			(No. Sterile/Total)			(No. Sterile/Total)	
			High Dose	Low Dose		High Dose	Low Dose		High Dose	Low Dose
24	2.0 ± 0.6	<LLOQ	2.8	4.5 *	39 ± 8.16	1.4	2.9	1134.4 ± 465.2	0	4.8
			(1.8–3.3)	(3.8–4.8)		(1.4–2.4)	(2.2–3.7)		(-)	(4.1–5.4)
			(2/9)	(0/6)		(6/9)	(0/6)		(7/7)	(1/5)
26	181.7 ± 38.5	44087 ± 37613.2	2.8	-	2232.2 ± 590.6	2.0	-	73509.4 ± 25767.2	0	-
			(1.8–3.6)			(1.4–2.7)			(-)	
			(1/9)			(5/9)			(8/8)	
28	81.9 ± 22.4	36688.8 ± 57680.9	2.7	-	2517 ± 1187.3	1.9	-	66556.3 ± 24606.8	0	-
			(1.5–4.3)			(1.1–4.3)			(-)	
			(2/9)			(0/9)			(7/7)	
32	37.1 ± 11.9	22844.9 ± 38340.4	2.1	-	809 ± 516.9	1.9	-	27835 ± 10871	0	-
			(1.8–3.4)			(1.1–3.9)			(-)	
			(1/8)			(5/8)			(4/4)	
48	2.6 ± 1	480.7 ± 319.5	2.3	3.7 *	44.9 ± 18.2	2.0	2.5	1380.6 ± 1096.9	2.0	2.6
			(1.9–3.4)	(2.9–4.1)		(0.8–3.3)	(2.1–4.1)		(-)	(1.0–2.9)
			(1/9)	(0/6)		(1/9)	(0/6)		(8/9)	(2/6)
72	1.1 ± 0	<LLOQ	3.1	4.0 *	31.2 ±0	2.2	2.4	1235.9 ± 894.3	3.1	2.4
			(2.0–3.4)	(3.3–4.1)		(1.3–2.5)	(1.4–3.1)		(2.0–3.5)	(2.0–2.8)
			(0/9)	(0/6)		(5/9)	(0/6)		(3/9)	(3/6)
Control	<LLOQ ^ϕ^	<LLOQ	2.9	4.3	<LLOQ	3.1	4.7	<LLOQ	2.9	3.2
			(1.7–4.1)	(4.0–4.4)		(2.3–4.3)	(3.3–5.5)		(2.3–3.1)	(3.0–5.8)
			(0/6)	(0/6)		(0/6)	(0/6)		(3/5)	(0/6)

^@^ Fosfomycin MIC for CFT073 *E. coli* is 64 µg/mL.# For urine samples, the number of animals varies from 1–9 based on the presence of urine in the urinary bladder at the time of necropsy.* ^ϕ^ LLOQ–Lower limit of quantification. *p* < 0.05, bladder median UPEC cfu for 24, 48, and 72 h high- and low-dose treatment groups; Student’s paired t-test.

**Table 4:** Fosfomycin long-term effect on viable organisms in the urinary tract tissues (urinary bladder, kidneys, and urine) of mice infected with *E. coli* strain CFT073.

Time (Weeks)	Days Post-Infection	Bacterial Counts (cfu/gm or mL) ^@^					
		Bladder		Kidneys		Urine ^#^	
		Median Log_10_ cfu/gm		Median Log_10_ cfu/gm		Median Log_10_ cfu/mL	
		Range		Range		Range	
		(No. Sterile/Total)		(No. Sterile/Total)		(No. Sterile/Total)	
		Treatment	Control	Treatment	Control	Treatment	Control
First week	Day 4	2.8	4.3 **	2.4	4.2	2	3.4
		(1.6–3.8)	(3.9–4.4)	(1.8–3.9)	(3.5–5.2)	(2.0–3.3)	(2.4–5.1)
		(0/6)	(0/6)	(0/6)	(0/6)	(0/5)	(0/6)
	Day 7	2.4	3.9	3.3	4.6	2.7	3.4
		(0.8–3.5)	(3.7–7.2)	(1.7–3.8)	(2.1–7.2)	(2.3–3.0)	(2.8–3.7)
		(0/6)	(0/6)	(0/6)	(0/6)	(0/2)	(0/5)
Second week	Day 4	2.9	3.0	1.5	2.2	0	3.1
		(2.5–3.1)	(2.5–3.0)	(1.4–1.5)	(1.4–3.3)	(-)	(3.1–3.2)
		(4/6)	(3/6)	(4/6)	(1/6)	(6/6)	(4/6)
	Day 7	2.3	2.5	1.4	1.6	0	2.4
		(0.5–2.6)	(2.1–2.9)	(1.3–2.0)	(1.1–1.6)	(-)	(-)
		(2/6)	(2/6)	(3/6)	(3/6)	(6/6)	(5/6)
Third week	Day 4	2.4	2.3	2	2.2	0	0
		(2.2–3.0)	(0.5–2.7)	(1.8–2.8)	(1.1–2.5)		
		(3/6)	(0/6)	(3/6)	(2/6)		
	Day 7	1.7	1.8	1.1	1.2	0	0
		(1.6–1.9)	(1.6–1.8)	(0.8–1.2)	(-)		
		(2/6)	(3/6)	(4/6)	(5/6)		

^@^ Fosfomycin MIC for CFT073 *E. coli* is 64 µg/mL.# For urine samples, the number of animals varies from 1–6 based on the presence of urine in the urinary bladder at the time of necropsy.** *p* < 0.01, bladder median UPEC cfu for first week day 4 treatment and control dose groups; Student’s paired t-test.

**Table 5:** Uropathogenic *E. coli* CFT073 strain–infected UTI mouse model treated orally with antibiotics and its resistant development at 1x minimum inhibitory concentration.

Treatment Group (Dose/kg)	1 × MIC Resistant Colonies
**Ampicillin**	MIC–4 µg/mL
High dose (200 mg/kg)	One urine sample in control group (1.4 × 10^3^ cfu/mL)
Low dose (20 mg/kg)	None
**Ciprofloxacin**	MIC–0.03 µg/mL
High dose (50 mg/kg)	One bladder (1.8 × 10^2^ cfu/mL) and one kidney (8.7 × 10^1^ cfu/mL) samples in 28 h. group;
	Two bladders (2 × 10^1^ and 7 × 10^0^ cfu/mL) and one kidney (4.26 × 10^2^ cfu/mL) samples in 32 h. group
Low dose (5 mg/kg)	One kidney sample each in 24 h. (1.3 × 10^1^ cfu/mL) and 72 h. (7 × 10^0^ cfu/mL) group
**Fosfomycin**	MIC–64 µg/mL
High dose (1000 mg/kg)	One urine sample in 24 h. (6.7 × 10^1^ cfu/mL) group
Low dose (100 mg/kg)	None
Long-term study (500 mg/kg)	One bladder (1 × 10^1^ cfu/ml) sample in day 4 of week 3
